# RETROSPECTIVE ANALYSIS OF THE IMPACT OF NEGATIVE PRESSURE WOUND THERAPY ON COMPLICATIONS OF DELAYED COVERAGE OF IIIB EXPOSED TIBIAL FRACTURES

**DOI:** 10.1590/1413-785220253303e290249

**Published:** 2025-12-01

**Authors:** Theodoro da Cunha Gonzalez, Ivan Ribaric, Yuri Macari Gomes, Mbilu Miguel André, Fernando Quissolo Dalaia Zua, Maria Adelaide de Miranda Gonçalves, Mauricio Ivo, Marcos de Camargo Leonhardt, Jorge dos Santos Silva, Kodi Edson Kojima

**Affiliations:** 1 Universidade de Sao Paulo, Faculdade de Medicina, Hospital das Clinicas (HCFMUSP), Grupo de Trauma, Instituto de Ortopedia e Traumatologia, Sao Paulo, SP, Brazil.

**Keywords:** Negative-Pressure Wound Therapy, Fractures, Ununited, Amputation, Tratamento de Ferimentos com Pressão Negativa, Fraturas não Consolidadas, Amputação

## Abstract

**Objective:**

To evaluate whether NPWT in delayed coverage of Gustilo-Anderson Type IIIB open tibial fractures reduces the complication rate, including deep infection, nonunion, and amputation.

**Methods:**

Retrospective case series including patients with Gustilo-Anderson Type IIIB open tibial shaft fractures treated between January 2014 and December 2017 with NPWT. The outcome measures were incidence of deep infection, nonunion, and amputation. Analysis of time to coverage (within 7 days vs. after 7 days) and type of soft tissue coverage.

**Results:**

26 patients with open tibial shaft fractures, predominantly male (96.2%), mean age 33.4 ± 13.9 years. Seven patients (26.9%) received coverage within 7 days; 19 patients (73.1%) received delayed coverage. The flap types were Myocutaneous (42.3%), microsurgical (34.6%), fasciocutaneous (23.1%). The overall infection rate was 50%, no significant difference between early (57.1%) and delayed (47.4%) coverage groups (p > 0.999). No amputations in the early coverage group; 2 (10.5%) in the delayed coverage group. Nonunion rates were 42.9% (early) and 36.8% (delayed). Overall complication rate: 85.7% (early) vs. 57.9% (delayed) (p = 0.357).

**Conclusion:**

NPWT may exert a protective effect in cases of delayed coverage by isolating the wound and promoting healing. *Level of Evidence III; Retrospective* ^f^ *study.*

## RESUMO

**Objetivo:**

Avaliar se a NPWT na cobertura tardia de fraturas abertas da tíbia tipo IIIB de Gustilo-Anderson reduz a taxa de complicações, incluindo infecção profunda, não união e amputação.

**Métodos:**

Série de casos retrospetiva incluindo pacientes com fraturas expostas da diáfise da tíbia tipo IIIB de Gustilo-Anderson tratados entre janeiro de 2014 e dezembro de 2017 com NPWT. As medidas de resultado foram a incidência de infeção profunda, não união e amputação. Análise do tempo para cobertura (dentro de 7 dias vs. após 7 dias) e tipo de cobertura de tecidos moles.

**Resultados:**

26 pacientes com fraturas expostas da diáfise da tíbia, predominantemente do sexo masculino (96,2%), com idade média de 33,4 ± 13,9 anos. Sete pacientes (26,9%) receberam cobertura em até 7 dias; 19 pacientes (73,1%) receberam cobertura tardia. Os tipos de retalho foram miocutâneo (42,3%), microcirúrgico (34,6%) e fasciocutâneo (23,1%). A taxa global de infecção foi de 50%, sem diferença significativa entre os grupos de cobertura precoce (57,1%) e tardia (47,4%) (p > 0,999). Não houve amputações no grupo de cobertura precoce; 2 (10,5%) no grupo de cobertura tardia. As taxas de não união foram de 42,9% (precoce) e 36,8% (tardia). Taxa global de complicações: 85,7% (precoce) vs. 57,9% (tardia) (p = 0,357).

**Conclusões:**

A NPWT pode proporcionar um efeito protetor nos casos de cobertura tardia, isolando a ferida e promovendo a cicatrização. *Nível de Evidência III; Estudio retrospectivo* ^f^.

## INTRODUCTION

Open tibial fractures are the most common type of open long bone fractures, typically resulting from a high energy trauma. These injuries are associated with significant muscle necrosis, high contamination and bone fragmentation leading to high morbidity and post-operative complications.^1,2^

The Gustilo-Anderson classification system is widely used to categorize open fractures based on the extent of soft tissue and bone damage, as well as the type and degree of contamination. Type III fractures involve multifragmentary fractures with extensive soft tissue damage; Type IIIB specifically includes cases with extensive periosteal stripping and exposed bone at the end of the procedure.^3^

Open fracture, comminuted fracture, infection and location of fracture in the tibia are among the most relevant risk factors contributing to fracture nonunion.^4^

Infection is the most devastating complication following an open tibial fracture due to its detrimental impact on clinical outcomes, burden to the healthcare system, chronic pain and psychological distress.^5^ Early soft tissue coverage is a critical factor in reducing the risk of infection, with earlier coverage correlating with lower infection rates.^6^

However, timely coverage is not always feasible, especially in cases of delayed in a severely contaminated wound which requires multiple debridement and many times the appropriately trained reconstructive surgeon is not available.^7^ Traditionally, wet-gauze saline dressings were applied to prevent wound exposure to nosocomial environments.^8^ However, since its approval by the Food and Drug Administration, negative pressure wound therapy (NPWT) has increasingly been used as an alternative dressing technique.

Although some studies have compared the outcomes of NPWT with conventional dressings, the superiority of NPWT in preventing complications has not been conclusively established.^9-11^

This study aims to evaluate whether the use of NPWT in delayed coverage of open tibial fractures classified as Gustilo-Anderson Type IIIB can reduce the complication rate.

## MATERIAL AND METHODS

This retrospective case series aimed to assess whether NPWT in delayed coverage of Gustilo-Anderson Type IIIB open tibial fractures decreases complications. The study included patients treated at a level one urban university trauma center between January 2014 and December 2017. The study was approved by the Research and Ethics Committee (approval number 10061019.0000.0068), and written informed consent was obtained from all participants.

The patient data were collected from the medical record and available radiographs and included age, sex, comorbidities, trauma mechanism, AO/OTA classification,^12^ type of fixation, type of definitive coverage, time between the injury and the definitive coverage and complications (infection, amputation and nonunion).

The inclusion criteria were patients over 18 years with open tibial shaft fracture Gustilo type IIIB who had been treated with NPWT, no other major injuries, minimum of 6 months of follow-up, and signed informed consent.

The exclusion criteria included open tibial shaft fracture IIIB treated with dressings other than the NPWT, type IIIB fractures in other bones, and polytrauma patients.

Initial treatment of open tibial fractures followed standard protocols, with thorough irrigation and debridement of all necrotic tissue.^7,11^ All fractures were fixed with an external fixator as part of a staged treatment approach. NPWT was applied using a polyurethane ether sponge with a pore size of 400–600 μm, covering the entire wound and sealed with an adhesive dressing. The system operated at 125 mmHg of continuous negative pressure. Definitive coverage was performed once the wound was clean, free of necrotic tissue, and a microsurgeon was available.

Complications were recorded, including deep infection as defined by Metsemakers et al.^13^ amputation and nonunion.

Qualitative patient characteristics were described using absolute and relative frequencies, while quantitative characteristics were summarized using mean, standard deviation, median, and quartiles. Outcomes were analyzed according to qualitative characteristics using Fisher's exact tests or likelihood ratio tests, and quantitative characteristics were compared using Student's t-tests or Mann-Whitney tests.14 Statistical analyses were conducted using IBM SPSS for Windows, version 22.0, and data were tabulated using Microsoft Excel 2013. A 5% significance level was used for all tests.^14^

## RESULTS

During the observation period (2014–2017), 41 patients with Gustilo-Anderson Type IIIB severe open fractures were treated, with 26 (63.4%) presenting with open tibial shaft fractures. Among these patients, 25 (96.2%) were male, with a mean age of 33.4 ± 13.9 years. Nine patients (34.6%) had comorbidities (Table 1).

**Table 1:** Patients’ demographics and outcomes.

Variable	Description (n = 26)
**Age**	
Mean ± SD Median (p25; p75)	33.4 ± 13.9 30 (22.8; 39.3)
**Sex, n (%)**	
Female Male	1 (3.8) 25 (96.2)
**Comorbidities, n (%)**	
Smoking Diabetes Alcoholism Illegal drugs	2 (7.7) 1 (3.8) 2 (7.7) 4 (15.4)
**Mechanism of injury**	
Motorbike accident Runover by car Car accident Crush injury	14 (53.8) 10 (38.5) 1 (3.8) 1 (3.8)
**Fracture AO classification, n (%)**	
Type A Type B Type C	12 (46.2) 8 (30.8) 6 (23.1)
**Soft tissue coverage, n (%)**	
Fasciocutaneous flap Myocutaneous flap Microsurgical flap	6 (23.1) 11 (42.3) 9 (34.6)
**Definitive fixation, n (%)**	
Intramedullary nail External fixation	17 (65.4) 9 (34.6)
**Time to coverage, n (%)**	
Within 7 days Over 7 days	7 (26.9) 19 (73.1)
**Complications, n (%)**	
Deep infection Nonunion Amputation	13 (50) 10 (38.5) 2 (7.7)

The most frequent mechanism of trauma was motorbike accident (53.8%) followed by pedestrian injury (38.5%).

The most prevalent AO/OTA fracture type was type A (46.2%), followed by the type B (30.8%) and type C (23.1%). All fractures were initially stabilized with an external fixator; 65.4% of fractures were subsequently fixed with an intramedullary nail, while 34.6% underwent a secondary external fixation.

All patients received intravenous antibiotics within 3 hours of injury and underwent irrigation and debridement within 12 hours. Seven patients (26.9%) received soft tissue coverage within 7 days, while 19 patients (73.1%) had delayed coverage beyond 7 days. Myocutaneous flaps were used for coverage in 11 patients (42.3%), microsurgical flaps in 9 (34.6%), and fasciocutaneous flaps in 6 (23.1%).

A total of 13 patients (50%) developed deep infections: 4 (57.1%) in the early coverage group and 9 (47.4%) in the delayed coverage group, with no statistically significant difference (p > 0.999). The infection rate showed no significant relationship with the type of fixation or the type of coverage.

No amputations occurred in the early coverage group, whereas 2 patients (10.5%) in the delayed coverage group required amputations. The nonunion rate was 42.9% in the early coverage group and 36.8% in the delayed coverage group. The overall complication rate was 85.7% in the early coverage group and 57.9% in the delayed coverage group (p = 0.357) (Table 2). Statistical analysis did not reveal any significant correlation between complications and demographic data (Table 3).

**Table 2:** Description of postoperative infection according to the characteristics assessed and results of statistical tests.

Variables	Deep infection		P
	No	Yes	
**Age (years)**			
Mean ± SD	30.8 ± 13.7	35.9 ± 14.2	0.355**
**Sex, n (%)**			
Female Male	1 (100) 12 (48)	0 (0) 13 (52)	>0.999
**Smoking**			
No Yes	12 (50) 1 (50)	12 (50) 1 (50)	>0.999
**Diabetes**			
No Yes	13 (52) 0 (0)	12 (48) 1 (50)	>0.999
**Alcoholism**			
No Yes	12 (50) 1 (50)	12 (50) 1 (50)	>0.999
**Illegal drugs**			
No Yes	11 (50) 2 (50)	11 (50) 2 (50)	>0.999
**Mechanism of injury**			
Motorbike accident Runover by car Car accident Crush injury	9 (64.3) 4 (40) 0 (0) 0 (0)	5 (35.7) 6 (60) 1 (100) 1 (100)	0.228#
**Fracture AO classification, n (%)**			
Type A Type B Type C	7 (58.3) 4 (50) 2 (33.3)	5 (41.7) 4 (50) 4 (66.7)	0.602#
**Soft tissue coverage, n (%)**			
Fasciocutaneous flap Myocutaneous flap Microsurgical flap	4 (66.7) 7 (63.6) 2 (22.2)	2 (33.3) 4 (77.8) 7 (77.8)	0.108#
**Definitive fixation, n (%)**			
Intramedullary nail External fixation	10 (58.8) 3 (33.3)	7 (41.2) 6 (66.7)	0.411
**Time to coverage, n (%)**			
Within 7 days Over 7 days	3 (42.9) 10 (52.6)	4 (47.1) 9 (47.4)	>0.999*

Chi square test;

* Fisher exact test; # likelihood ratio test;

** unpaired Student t test.

**Table 3:** Description of all post operative complications (infection, amputation and nonunion) according to the characteristics assessed and results of statistical tests.

Variables	All complications		p
	No	Yes	
**Age (years)**			
Mean ± SD	32.7 ± 15.3	33.7 ± 13.6	0.860**
**Sex, n (%)**			
Female Male	1 (100) 8 (32)	0 (0) 17 (68)	0.346
**Smoking**			
No Yes	9 (36) 0 (0)	15 (62.5) 2 (100)	0.529
**Diabetes**			
No Yes	9 (36) 0 (0)	156 (64) 1 (100)	>0.999
**Alcoholism**			
No Yes	9 (37.5) 0 (0)	15 (62.6) 2 (100)	0.529
**Illegal drugs**			
No Yes	8 (36.4) 1 (25)	14 (63.3) 3 (75)	>0.999
**Mechanism of injury**			
Motorbike accident Runover by car Car accident Crush injury	7 (50) 2 (20) 0 (0) 0 (0)	7 (50) 8 (80) 1 (100) 1 (100)	0.248#
**Fracture AO classification, n (%)**			
Type A Type B Type C	4 (33.3) 3 (37.5) 2 (33.3)	8 (66.7) 5 (62.5) 4 (66.7)	0.979#
**Soft tissue coverage, n (%)**			
Fasciocutaneous flap Myocutaneous flap Microsurgical flap	3 (50) 4 (36.4) 2 (22.2)	3 (50) 7 (63.6) 7 (77.8)	0.530#
**Definitive fixation, n (%)**			
Intramedullary nail External fixation	7 (41.2) 2 (22.2)	10 (58.5) 7 (77.8)	0.418
**Time to coverage, n (%)**			
Within 7 days Over 7 days	1 (14.3) 8 (42.1)	6 (85.7) 11 (57.9)	0.357*

Chi square test;

* Fisher exact test; # likelihood ratio test;

** unpaired Student t test.

## DISCUSSION

Open tibial shaft fractures are the most common open long bone fractures, accounting for 11% of all open fractures.^15^ These injuries are often caused by high-energy trauma, leading to a high incidence of severe fractures, such as Gustilo-Anderson Type IIIA and IIIB. Chua et al. reported that 52.6% of these fractures are classified as Gustilo-Anderson Type IIIA and IIIB, with Type IIIB accounting for approximately 20% of all Type III fractures, or about 10% of all open tibial shaft fractures.^16^

To minimize the risk of infection in Gustilo-Anderson Type IIIB fractures, which are associated with significant soft tissue loss, periosteal stripping, and exposed bone, early antibiotic administration, thorough irrigation and debridement, skeletal fixation, and soft tissue management are critical.^17^Godina advocated for early soft tissue reconstruction to reduce complications.^18^

However, early coverage is not always feasible due to factors such as severe contamination, difficulty in defining debridement margins, and the unavailability of a trained reconstructive surgeon. NPWT can serve as adjunctive therapy to protect the wound in such cases.^19^

The total number of patients requiring staged treatment for Type IIIB open tibial fractures is relatively low. Mathieu et al ^20^eported 5.4 per year, Cullen et al.^21^ 9.4 per year, Bhattacharyya et al.^22^ 9.5 per year and Gopal et al.^23^ 9.3 per year. Our study observed an average of 6.5 cases per year, totaling 26 patients, similar to the published articles. This small sample size limits the statistical power of the study and the applicability of its conclusions.

he demographics of our patient cohort align with other studies, consisting primarily of young male adults involved in road traffic accidents with relatively simple fractures (Table 1).^24^ The use of flaps reflects the severity of soft tissue injuries, with 42% requiring myocutaneous flaps and 35% requiring microsurgical flaps. In our study, 26.9% of patients received wound coverage within 7 days, while 73.1% had delayed coverage.

Comparisons with the literature are challenging due to the variability in reported infection rates, which range from as low as 1.5% in Godina^18^ study, to 19% with Kumaar,^25^ to around 30% with Cullen^21^ and Singh,^26^ to as high as 50% to 60%, respectively by Bhattacharyya^22^ and Mathieu,^20^ This variation likely reflects the heterogeneity of Gustilo-Anderson Type IIIB injuries, which can range from minor to extensive lesions.

Although it is generally expected that delayed coverage increases the complication rate, our study found similar infection rates in both early (47.1%)^24^ and delayed coverage groups (47.4%).^25^ The use of NPWT may have mitigated the risk of infection in delayed cases by isolating the wound, removing secretions and infectious material, and promoting neovascularization.^19^

The nonunion rate was 38.5%, with 2 amputations (7.7%), showing no significant difference between the early and delayed coverage groups. Thus, NPWT may offer a protective effect in cases of delayed coverage, reducing the risk of nonunion and amputation.

This study has some limitations. The small sample size may affect the statistical power of the association and results. However, as mentioned before, the number of type IIIB open tibia shaft fracture with need for flap is low. The retrospective nature of the study has the disadvantage of this type of design. And, besides the NPWT other factors may play a role in the development of infection, like revision surgery, multiple debridement, type of antibiotics, time to surgery, etc.

We recommend conducting a prospective randomized multicenter study with a larger number of patients in each group, and control of the confound variables to get more accurate statistical results.

## CONCLUSION

The use of NPWT in Gustilo-Anderson Type IIIB open tibial fractures may help prevent an increase in complications such as deep infection, nonunion, and amputation, even in cases of delayed coverage. Given the severity of these injuries, the complication rate remains high, but NPWT may help maintain the complication rate in cases where coverage is delayed.
